# How to interpret and integrate multi-omics data at systems level

**DOI:** 10.1080/19768354.2020.1721321

**Published:** 2020-01-30

**Authors:** Gun Tae Jung, Kwang-Pyo Kim, Kwoneel Kim

**Affiliations:** a Department of Biomedical Science and Technology, Kyung Hee University, Seoul, Republic of Korea; b Department of Applied Chemistry, Kyung Hee University, Yongin, Republic of Korea; c Department of Biology, Kyung Hee University, Seoul, Republic of Korea

**Keywords:** Multi-omics, co-expression network, transcriptional regulatory network, protein interactome network, transcriptional core regulator

## Abstract

Current parallel sequencing technologies generate biological sequence data explosively and enable omics studies that analyze collective biological features. The more omics data that is accumulated, the more they show the regulatory complexity of biological phenotypes. This high order regulatory complexity needs systems-level approaches, including network analysis, to understand it. There are a series of layers in the omics field that are closely connected to each other as described in ‘central dogma.’ We, therefore, have to not only interpret each single omics layer but also to integrate multi-omics layers systematically to get a full picture of the regulatory landscape of the biological phenotype. Especially, individual omics data has their own adequate biological network to apply systematic analysis appropriately. A full regulatory landscape can only be obtained when multi-omics data are incorporated within adequate networks. In this review, we discuss how to interpret and integrate multi-omics data systematically using recent studies. We also propose an analysis framework for systematic multi-omics interpretation by centering on the transcriptional core regulator, which can be incorporated in all omics networks.

## Introduction

Nowadays, an enormous amount of sequencing data has been generated by increasing levels of size exponentially. Next-generation sequencing (NGS) technology is a key breakthrough that performs parallel sequencing with fewer specimen than traditional sequencing methods. The old gene studies focused on a set of genes within a couple of samples, while current studies profile whole genome from a myriad of samples. We call this genomics, which indicates the study of collective features of whole genomes as described by its suffix ‘-ome,’ meaning ‘totality.’ Recent genomics studies have unraveled mechanisms underlying complex phenotypes or diseases such as cancer, which had not yet been well understood. Especially, large-scale genomics have revealed that most complex traits have polygenic loci on human genomes in regard to complex diseases (Torkamani et al. 2018). Furthermore, it has been reported that the association landscape of genomic loci are shown to be ‘omnigenic’ for complex traits (Boyle et al. 2017), which implies that all the loci incorporate in the regulation of relevant phenotypes. This high order regulatory complexity needs systems-level understanding that comprises network analysis.

Not only has genomics, but also other omics, evolved along with the development of technologies related to cellular and molecular biology, such as reverse transcriptase PCR (RT-PCR), chromatin immunoprecipitation (ChIP), and mass spectrometry (MS). These experimental technologies have been incorporated into NGS accordingly by developing transcriptomics, epigenomics, and proteomics, although proteomics requires a particular process (Cravatt et al. 2007). Each omics can be used to explain only biological phenomena specific to its own omics field. Therefore, scientists have attempted to integrate individual omics layers to unravel overall mechanisms that are obscure in a single omics study. We called this integrative work a multi-omics study. In this review, we summarize recent multi-omics studies that try to converge two or more omics fields at the systems-level to address complex biological mechanisms difficult to understand using current technology.

## Results

### Interpretation of genomic variants with chromatin long-range interaction

Most transcriptional regulations have been revealed to have a couple of regulators targeting a set of genes. This regulatory architecture is constructed by promoter-enhancer interactions. We can map where transcription factors bind in the genome using chromatin immunoprecipitation sequencing (ChIP-seq) (Johnson et al. 2007) and determining where these transcription factors regulate a specific gene using long-range interaction experiments such as Hi-C (Lieberman-aiden et al. 2009) and ChIA-PET (Fullwood and Ruan 2009) technologies. Epigenetic data produced by the above technologies were integrated with the data of genomic variations to interpret their functionality for human genomes, and it was revealed that not only were there variants in coding regions, but also variants in noncoding regions that were associated with human disease development through the perturbation of transcriptional regulation. For example, recurrent mutations on the promoter of the TERT gene generated ETS factor binding sites and fluctuated TERT expression, factors that encode a catalytic subunit of the enzyme telomerase so that the risk of melanoma development increased because cell fates were made instable (Huang et al. 2013). In another example, the ETV1 promoter is known for its interacting enhancers that affect ETV1 expression, which influences cell viability and patient survival for colorectal cancer (Feigin et al. 2017). Both studies on cancer and a study on Hirschsprung disease have shown that variants on multiple enhancers act to increase disease risk by driving the dysfunction of the gene regulatory network (Chatterjee et al. 2016). These studies imply that there are complex genetic interactions whose variations drive disease development, which are revealed by integrative studies addressing genomic and epigenome data.

In our previous study, we developed a prediction model to identify candidates for cancer driver genes by leveraging a variety of genomic and epigenome data in the context of transcriptional regulation (Kim et al. 2016). The epigenome data for chromatin long-range interactions was critical in improving sensitivity to identify driver mutations. The prediction model successfully predicted and validated the functionality of TERT recurrent mutations for lung cancer, which has not been previously reported. Moreover, recent progressive studies have shown that combinatorial and nonlinear modeling of genomic and epigenomic patterns shared by risk variants have successfully predicted potential causal variants in major psychiatric disorders and autoimmune diseases (Lee et al. 2019). The deep learning algorithm convolutional neural network was used to construct a prediction model by using over 2,000 functional features that were mainly about genetic and epigenetic characteristics such as histone modifications, chromatin accessibility, transcription factor binding, and target gene function. The predicted causal variants in this study were enriched in active regulatory regions that contained binding sites of transcription factors of the relevant cell type. Furthermore, they resulted in the expression alteration of genes associated with the given disease. The two aforementioned machine learning-based approaches imply that genetic and epigenetic features actively associated in nonlinear levels to shape the causal regulatory interactions of complex genetic diseases.

### Transcriptional regulatory network incorporating an epigenomic landscape

To unravel the transcriptional regulatory complexity across a set of samples, weighted gene co-expression network analysis (WGCNA) has been used based on pairwise correlations between gene expression variables (Langfelder and Horvath 2008). This method defines several co-expression subnetworks so that a distinct feature for each subnetwork can be studied independently and compared for their topologies. Furthermore, more accurate network interactions have been inferred by an algorithm called the reconstruction of accurate cellular networks (ARACNe) (Margolin et al. 2006), which eliminates the majority of indirect interactions constructed by the WGCNA method. ARACNe is used as a data reduction technology to make co-expression networks suitable for integrating complementary biological data. The integrative analyses were proposed to perform a systems study based on co-expression networks (van Dam et al. 2018). Pathway enrichment analysis can be incorporated to reveal specific biological pathways relevant to a co-expression subnetwork. In addition, pathway enrichment analysis is able to determine which samples contribute to the construction of the subnetwork by calculating eigenvector during the co-expression network construction procedure. These methods of analyses help to define a candidate function for the target subnetwork or subgroup that we are interested in. For example, an integrative network study using the above methodologies successfully identified MYC as a major hub that controls a transcriptional regulatory network in human B cells (Basso et al. 2005).

However, the above methodologies have not been capable of inferring a causal relationship indicating regulatory direction, although previous network analyses have been so effective in studying transcriptional regulation. In a series of studies, the binding of transcription factors has been shown to be a critical explanatory factor in the causality of transcriptional regulation. The landscape of transcription binding patterns has formed a regulatory hierarchy that governs a group for gene expression and disease development (Wang et al. 2007). In addition, a set of transcription factors have been shown to maintain human or mouse embryonic stem (ES) cells in a pluripotent state by binding to the promoter of their target genes cooperatively (Kim et al. 2008). Furthermore, the dynamics of human transcription factor binding and regulation have been analyzed at the systems-level comprehensively for over 400 transcription factors across over 40 cell and tissue types (Neph et al. 2012). In this study, transcription factor regulatory networks were revealed to be highly cell selective and driven by subsets of transcription factors that have roles in the control of cellular identity. These studies have demonstrated that transcription factor machinery binds to regulatory regions to cause the regulation of target genes resulting in the development of cells and diseases. Because the transcription factor machinery determines the transcriptional regulatory direction, it can be used to construct a framework for the full picture of transcriptional regulation. In our previous study, we developed a Bayesian probabilistic model by using promoter-enhancer interactions and relevant transcription factor binding data in a breast cancer cell. A transcriptional regulatory network with causal interactions was then constructed accordingly using machine learning for gene expressions in breast cancer patients (Kim et al. 2015). We unraveled the regulatory complexity underlying tumor subclasses and drug responses by using these causal relationships. This systematic modeling of epigenomic regulations coupled with machine learning of transcriptomic variables was critical in determining the true biological interactions with increased overall coverage and specificity.

### Multi-omics approaches center on the proteome

Genomes, epigenomes, and transcriptomes can be sequenced by NGS technology, whereas proteomes cannot be sequenced by NGS and can only be sequenced by mass spectrometry (MS) directly (Cravatt et al. 2007). It is more arduous to quantify and normalize output sequences in MS technology than NGS. Therefore, in recent studies, genomics approaches have been used as a blueprint for the accurate mapping of proteomics sequences, which is referred to as proteogenomics (Nesvizhskii 2014). The regulatory landscape of the proteome is also different from the regulations that occur in genomes, epigenomes, and transcriptomes. Transcription factor binding to the regulatory regions of genomes is a key factor across the regulation of genomes, epigenomes, and transcriptomes; however, a physical interaction between proteins is crucial in the regulation of proteomes. We refer to this physical interaction as protein–protein interaction (PPI). A myriad of PPIs perform critical roles in cell signaling, pathways, and transcription factor co-binding. Yeast two-hybrid screening (Y2H) is widely used to reveal this interaction (Hurt et al. 2003), which is a molecular biology experiment that tests whether two compartments of transcription factors successfully activate a target gene. The two compartments are the DNA-binding domain (DBD) and activation domain (AD) so that they can activate transcription of the target gene only when they functionally interact with each other. Currently, a systematic network of ∼14,000 human binary PPIs have been published as a reference map for protein interactome networks, which helps to understand the functional relationships at the proteome level (Rolland et al. 2014). The human protein interactome network has been updated recently; therefore, we can integrate interactome network information into current transcriptional regulatory networks to expand the regulatory network.

In addition to the sequencing difficulty of proteomics, another crucial factor making MS-based proteomics difficult is post-translational modification (PTM). PTM is a series of protein modification processes, such as the addition or removal of chemical moieties of amino acids, changes in protein properties caused by proteolytic processing, and the formation of disulfide bridges between cysteine residues (Mann and Jensen 2003; Larsen et al. 2006). PTM contributes to a variety of biological processes including the regulation of metabolism and cellular signaling events. Advancements in MS-based proteomics technology, especially coupled with high-performance liquid chromatography, have been used in the characterization of PTMs (Jensen 2004; Larsen et al. 2006), and a number of PTMs have been identified in eukaryotic proteins (Csizmok and Forman-Kay 2018). A recent study was conducted by integrating proteome and PTM data coupled with transcriptome profiling to reveal the molecular heterogeneity within early-onset gastric cancers (EOGCs) (Mun et al. 2019). The four subtypes of EOGCs that include proliferation, immune response, metabolism, and invasion were identified by analyzing the transcriptome, global proteome, phosphoproteome, and N-glycoproteome. These subtyping results could help to develop strategies for patient stratification and treatment. However, there are few systematic studies for multi-omics approaches because the characteristics between proteomes and other omics are considerably different in network biology. A progressive study reported multi-omics approach centered on proteomics at the systems level. In the study, deep profiling of whole proteomes, phosphoproteomes, and transcriptomes was performed in glioma mouse models (Wang et al. 2019). Consequently, systems analysis for this multiple omics data revealed master regulators, including kinases, and transcription factors to explain glioma drivers. This study comprised a pathway analysis of an integrative network that was composed of proteomic, phosphoproteomic, and transcriptomic interactions. The integrated network approaches extended beyond simple identification of pathways as they were constructed of a single type of omics data. These studies imply that proteomics-centered approaches can bring new insights on the regulation of protein activities that advance current understandings of the transcriptional regulatory landscape. Furthermore, the more layers of omics data that are added, the more easily we will obtain a full picture of the regulatory landscape of biological phenomena.

### A proposal for integrated multi-omics analysis at the systems level

**Figure 1.**
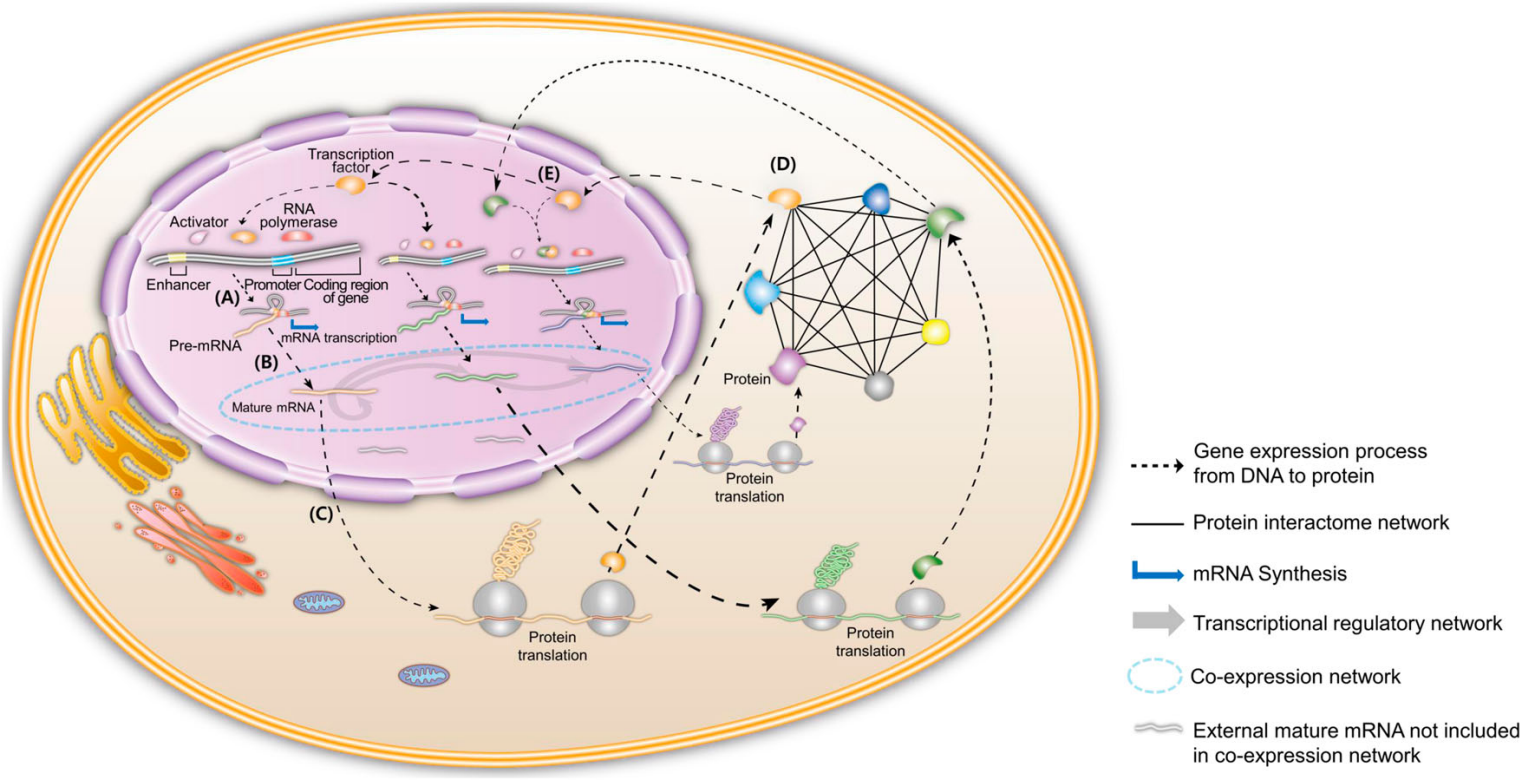
Diagram for integrative network modeling for multi-omics data according to the gene expression process. (A) Transcription factors bind to cis-regulatory regions, such as the promoter and enhancer of DNA, and RNA polymerase is attached to the promoter to form an initiation complex and synthesize pre-mRNA. (B) Pre-mRNA undergoes further procedures to become mature mRNA and co-expression networks (blue dotted ellipses) that are formed if there are significant expression correlations between transcripts. This co-expression network can be constructed by using transcriptome data. (C) mRNA transfers from the nucleus to the cytoplasm and binds to ribosomes to synthesize proteins. (D) Synthesized proteins form networks of protein complexes through protein–protein interactions (black solid line). This interactome network can be analyzed based on proteome data. (E) Some transcription factors are transported into the nucleus and then regulate their target genes by binding cis-regulatory regions. In this figure, the transcription factor colored by orange binds all regulatory regions below target genes colored by orange, green, and purple. The orange transcription factor can regulate all downstream genes at the transcriptional regulatory level; we called this the transcriptional core regulator. This regulatory landscape composes a transcriptional regulatory network (grey arrow) that can be constructed using epigenome data.

Based on the above multi-omics studies and systems approaches, we propose a comprehensive systems model to integrate each multi-omics layer according to the gene expression process (Figure 1). First, a co-expression network is constructed to build a framework accommodating transcriptional regulations by using transcriptome data. This allows a myriad of genes that are difficult to understand simultaneously to be divided into subnetworks that are coregulated together at the transcriptome level. It can be unraveled how the subnetwork is involved in a specific biological mechanism when pathway analysis is performed for the relevant subnetwork. After that, the epigenomic regulatory landscape, including transcription factor binding, chromatin long-range interaction, and chromatin accessibility, is used to infer the regulatory direction (Kim et al. 2016). We then obtain a set of subnetworks, which are related to specific pathways, and have concrete regulatory directions. By analyzing these subnetworks by tracing back the regulatory directions, the core transcriptional regulator existing at the top of the subnetwork topology can be discovered. We can subsequently identify the core regulator’s functionality with respect to a specific phenotype through network analysis. As an example of this analysis, differentially expressed genes (DEGs) between two groups having a difference for an interesting phenotype can be mapped to the co-expression subnetworks. There would be one or more pathway and core regulator for each subnetwork that is mapped by DEGs. We then detected several candidate subnetworks comprising pathways that explain the interesting phenotype properly. Furthermore, the connectivity between the core regulators and the candidate subnetworks can be calculated according to how many DEGs are included under the regulation of the core regulator. This indicates that the core regulator that is connected more to the candidate subnetwork can better explain about the expression alteration for the interesting phenotype.

However, network analysis based on transcriptional regulation cannot fully explain phenotypic alteration systematically. Many crucial alterations for phenotypic regulation occur at the protein level. Therefore, when it is hard to find significant DEGs in the context of transcriptional regulation, differentially expressed proteins (DEPs) based on proteomic analysis can offer another key regulatory explanation. The transcription factor, chromatin modifier, and other epigenomic regulators are core regulators in transcriptional regulatory networks, which are composed of proteins. This implies that variations in these regulators at the protein level are also important to understanding the regulatory mechanisms of regulatory networks. Not only is profiling DEPs themselves important, but analyzing PTM will give us additional information for multi-omics systems analysis. Moreover, PPI networks can supplement and enhance transcriptional regulatory networks by attaining gene-level signatures from profiling results of proteins and PTMs to construct integrative regulatory networks. For example, when we discovered that transcription factor A regulates a set of genes with DEGs or DEPs, which have functional enrichment relevant to an interesting phenotype such as a specific disease, the alteration of transcription factor A can be crucial in explaining the marker for the development of the disease. However, there may be no alteration for transcription factor A at the (epi)genomic, transcriptomic, and proteomic levels in another cohort, although it ranks highest among candidates for markers of the disease. In this case, protein interactome analysis specifically can help to determine another factor that affects transcription factor A in interaction regulation. This is because alterations of other co-regulating factors that interact with transcription factor A might influence transcriptional regulation. By integrating this interactome network information with the constructed network, we can get a full regulatory map from transcription to translation for a specific phenotype.

## Conclusion

To understand regulatory mechanisms in a complex biological system, all layers of omics that include genomics, epigenomics, transcriptomics, and proteomics are necessary as they play independent but related roles in relation to one another. In addition, considerable expressions of mRNA do not always result in protein expressions, although large portions of mRNA production are correlated with protein levels (Liu et al. 2016). This is because a variety of mRNA outputs are associated with the regulation of a set of proteins rather than a correspondence between the regulation of mRNAs and proteins that is one-to-one. Therefore, we should not only profile each layer of omics, but also analyses all the layers simultaneously at the systems level to obtain a full picture of actual gene regulation. The term ‘Trans-Omics’ has been proposed, which is the reconstruction of a global network across multiple omics layers by using multi-omics measurements and data integration (Yugi et al. 2016).

There is another omics layer to enhance the resolution of a full regulatory picture that is metabolomics. This layer has completely different characteristics compared to other omics layers as explained previously. Metabolomics addresses chemical processes involving metabolites from cellular processes so that it covers the latest step of the whole multi-omics procedures. Metabolomics provides a direct physiological state for an active cellular function, although analytical technologies such as MS or nuclear magnetic resonance (NMR) (Dettmer et al. 2007) are more difficult to interpret than proteomics approaches. The analysis results for metabolomics is substantially different according to the analyzed tissue and time due to the metabolomic dynamics of a living cell, which makes it difficult to conduct robust analyses. Nevertheless, the technology for metabolomics is developing gradually and has recently become an essential omics layer to study multi-omics. In addition to this supplementary layer for multi-omics study, additional interaction information can enhance the capacity of regulatory networks systematically. One example of a human functional gene network called HumanNet, which integrates a series of omics data using Bayesian statistics, allows for more flexible incorporation of network information into studies (Hwang et al. 2019). This network analysis is capable of extending or validating the existent network biology. One of our recent studies proposed that the extension of transcriptional drivers using both of physical and functional interactome networks successfully identified known coding drivers in cancer (Jang et al. 2017).

Taken together, a series of omics data using the relevant network construction will enhance our understanding of complex mechanisms that underly biological phenotypes when the multi-omics data are incorporated within an adequate framework. The framework should be constructed based on ‘central dogma,’ which indicates information flow from transcription to translation. Each step of making mRNAs and proteins is composed by complex networks, and they interact with each other. Therefore, systems approaches that use transcriptional regulatory network, protein interactomes, and functional networks have to be incorporated properly to the relevant omics data. In addition, we propose analysis that identifies a core regulator of transcriptional regulatory networks. This transcriptional core regulator-centered approach has the advantage in multi-omics studies because the perturbation of the core regulator can be affected by or affect not only transcription but also translation. Most of the core regulators may be transcription factors so that they actively participate in transcriptional regulation. Furthermore, they are associated with translation as they are composed of proteins. Therefore, core regulators can be incorporated in both of transcriptional regulatory networks and protein interactomes. Systematic multi-omics interpretation in the context of perturbation of transcriptional core regulators will successfully give us clues to unravel mechanisms about complex biological phenotypes.
